# Family-based germline sequencing in children with cancer

**DOI:** 10.1038/s41388-018-0520-9

**Published:** 2018-10-10

**Authors:** Michaela Kuhlen, Julia Taeubner, Triantafyllia Brozou, Dagmar Wieczorek, Reiner Siebert, Arndt Borkhardt

**Affiliations:** 1 Department of Pediatric Oncology, Hematology and Clinical Immunology, University Children’s Hospital, Medical Faculty, Heinrich Heine University, Düsseldorf, Germany; 2 Institute of Human Genetics, Medical Faculty, Heinrich Heine University, Düsseldorf, Germany; 3 Institute Human Genetics, Ulm University & Ulm University Medical Center, Ulm, Germany

**Keywords:** Paediatric cancer, Medical genetics

## Abstract

The discovery of cancer-predisposing syndromes (CPSs) using next-generation sequencing (NGS) technologies is of increasing importance in pediatric oncology with regard to diagnosis, treatment, surveillance, family counselling and research. Recent studies indicate that a considerable percentage of childhood cancers are associated with CPSs. However, the ratio of CPSs that are caused by inherited vs. de novo mutations (DNMs), the risk of recurrence, and even the total number of genes, which should be considered as a true cancer-predisposing gene, are still unknown. In contrast to sequencing only single index patients, family-based NGS of the germline is a very powerful tool for providing unique insights into inheritance patterns (e.g., DNMs, parental mosaicism) and types of aberrations (e.g., SNV, CNV, indels, SV). Furthermore, functional perturbations of key cancer pathways (e.g., TP53, FA/BRCA) by at least two co-inherited heterozygous digenic mutations from each parent and currently unrecognized rare variants and unmeasured genetic interactions between common and rare variants may be a widespread genetic phenomenon in the germline of affected children. Therefore, family-based trio sequencing has the potential to reveal a striking new landscape of inheritance in childhood cancer and to facilitate the integration and efforts of individualized treatment strategies, including personalized and preventive medicine and cancer surveillance programs. Consequently, cancer genetics is becoming an increasingly common approach in modern oncology, so trio-sequencing should also be routinely integrated into pediatric oncology.

## Introduction

Lifestyle factors such as UV exposure, smoking and alcohol consumption are major contributors to cancer development in adults. As these factors are negligible in children, one can speculate that a substantial (and previously underestimated) number of pediatric cancers must be attributable to inherited mutations in cancer predisposition genes (CPGs), currently unrecognized rare variants, and the combination of inherited susceptibility and environmental factors such as influences during pregnancy and infection exposure [1]. Recent studies indicate that 8.5% of childhood cancers are associated with cancer predisposition syndromes (CPSs), including 16.7% of non-central nervous system solid tumors, 8.6% of central nervous system tumors and 4.4% of leukemias [2]. In fact, it is probable that the percentage of cancers linked to CPSs in children is even higher. In a recent pan-cancer study by the International Cancer Genome Consortium, likely deleterious variants of 109 known autosomal CPGs were shown to affect 11% of 2642 cancer patients across 39 cancer types. This number increased to 20% of donors when considering germline pathogenic variants in 183 DNA damage-response genes, which do not have a presently established link to cancer risk [3]. However, the exact proportion of children and adolescents with a malignancy that is attributable to an underlying CPS is still unclear. A major reason for this is the fact that most published data on this topic relies on sequencing of only index patients, i.e., the affected children. These data does not take into account the family context, and, therefore, valuable discovery and interpretation information are disregarded. The most well-known mutated genes in childhood cancer are *TP53*, followed by *APC, BRCA2, NF1, PMS2, RB1*, and *RUNX1* [2]. According to a recent study, affected families show great interest in genetic testing for an underlying CPSs [4].

Unexpectedly, the predictive value of the family history is still unclear, as related studies report inconsistent results [2, 5]. Additionally, the proportion of de novo vs. inherited germline mutations in CPGs is widely undetermined resulting in considerable uncertainty about recurrence risk in siblings. For example, the prevalence of *TP53* mutations has been estimated to be anywhere from 1 in 20,000 up to 1 in 5000, with 7–24% being expected to occur de novo [6]. In contrast, ~50% of the mutations in *NF1* originate de novo [7].

The identification of children affected with CPSs could have direct impact on therapeutic cancer management. For instance, Li–Fraumeni syndrome (LFS) patients have an increased risk of radiation-induced secondary malignancies [8].

## Next-generation germline sequencing of parent-child trios

Genetic variations arise through new mutations; thus, determining the properties and rates of mutations is fundamental to understanding the genetics of human disease. Due to technical limitations, the number of loci studied was limited in past mutation rate analyses. However, advances in sequencing technology rapidly replaced classic molecular diagnostics, and the number of its applications has increased immensely in the past decade. Next-generation sequencing (NGS) provides a powerful tool to identify genomic variations associated with specific diseases, including cancer.

With increasing adoption of whole-exome sequencing (WES) and whole-genome sequencing (WGS), the detection of novel, previously uncharacterized sequence variants has increased and will continue to increase dramatically in the near future. Today, using NGS approaches, the occurrence of all types of mutations, including single-nucleotide variants (SNVs), small insertions and deletions (indels) and also large structural variations (SVs) can be analyzed. Compared to WES, WGS is the better technique to detect many types of variants, including indels, non-coding variants, CNVs, repeat expansions, and SVs (such as inversions and translocations) and can also reveal pathogenic mutations in the non-coding part of the genome (promoter regions, introns, enhancer and regulatory regions). However, both methods are hampered by challenges in methodical approaches (e.g., depth, coverage), data analysis and interpretation, storage of vast amounts of data, and relatively high costs.

Typically, in cancer syndromes only the single patient is sequenced. However, in order to test hereditary CPSs and family members at high-risk, WES of parent-child trios has become an increasingly popular strategy. In children with rare diseases, particularly in the field of intellectual disability (ID), autism spectrum disorder (ASD) and primary immunodeficiency, this strategy allows identifying causative genetic variants [9–12]. Lee et al. very nicely demonstrated with a diagnosis rate of 31% in 410 undiagnosed children with suspected genetic conditions, that trio clinical exome sequencing (CES) is superior to proband-CES only (diagnosis rate 22%, *p* = 0.002) and effectively detects *de novo* and compound heterozygous variants [13]. This finding was subsequently confirmed by Farwell et al. with a diagnostic rate of 37% in a family-based exome sequencing approach as compared to 21% with a singleton testing strategy and furthermore in a meta-analysis performed by Clark et al. (odds ratio 2.04, 95% CI: 1.62-2.56, I(2) = 12%; *P* < 0.0001) [14]. In addition, Mestek-Boukhibar et al. reported the development of a comprehensive real-life workflow for the use of trio WGS in critically ill children with a molecular diagnosis in 42% children, in 30% of these with immediate impact on clinical management [15]. An overview of studies based on trio sequencing is given in Table 1.

**Table 1:** Overview on Trio-NGS Studies

Research of the study	Sample size	Tissue type	Type of sequencing (WGS or WES)	Analysis pipeline	Silent mutations	Main research findings	Reference
Determination of the contribution of post-zygotic events to *de novo* mutations and embryonic mosaicism. Study analyzes 107 d*e novo* mutations in 50 trios	50 parent-child trios	Peripheral blood	whole-genome sequencing (80-fold coverage for defining *de novo* mutations, PCR amplicons for amplicon-based deep sequencing (ADS) and sanger sequencing for post-zygotic state of *de novo* mutations)	Variants were called with CG software v.2.4. *de novo* mutations were called with CG’s cgatools calldiff program	not considered	6.5 % of the presumed germline *de novo* mutations were in fact present as mosaic mutation in the blood of the child and were therefore, likely to have occured post-zygotically, important fraction of *de novo* mutations presumed to be germline and occured either post-zygotically in the child or were inherited as a consequence of low-level mosaicism in one of the parents	[50]
National study of the human genome in order to discover a complete set of variations between individual genomes. Trio approach to identify *de novo* mutations and develop a probabilistic method to determine *de novo* mutation rates for SNVs and indels	10 parent-child trios	Peripheral blood	whole-genome sequencing (high depth, 50×)	SNVs and short indels were called using the genome analysis toolkit (GATK)	not considered	They reproted 536k novel SNVs and 283k novel short indels from mapping approaches and developed a population-wide *de novo* assembly approach to analyse 132k novel indels larger than 10 nt with low false discovery rates. They used trio information to identify d*e novo* mutations and used a probabilistic method to provide direct estimates of 1.27e-8 and 1.5e-9 per nucleotide per generation for SNVs and indels	[79]
The first direct comparative analysis of male and female germline mutation rates from the complete genome of two parent-child trios	2 parent-child trios	Peripheral blood	whole-genome sequencing	Three different algorithms, the family-aware probabilistic Illumina-read–based method, the family-aware Illumina genotype-likelihood–based method and the sample-independent multiple technology genotype–based method were developed for DNM discovery	not considered	Identification of 49 and 35 germline *de novo* mutations (DNMs) in two trios, in one family 92% of germline DNMs were observed from the paternal germline, whereas in contrast in the other family 64% of DNMs were from the maternal germline. These observations suggest considerable variation in mutation rates within and between families	[53]
Diagnostic exome sequencing was immediately successful in diagnosing patients in whom traditional technologies were uninformative. The data demonstrate the utility of family-based exome sequencing and analysis to obtain the highest reported detection rate in an unselected clinical cohort, illustrating the utility of diagnostic exome-sequencing as a transformative technology for the molecular diagnosis of genetic disease	500 parent-child trios	Peripheral blood	whole-exome sequencing	The sequence data were aligned to the reference human genome (GRCh37), and variant calls were generated using CASAVA (Consensus Assessment of Sequence And Variation, Illumina) and Pindel. The HGMD, the Single Nucleotide Polymorphism database, the 1000 Genomes Project,HapMap data, and online search engines like PubMed were used to search for previously described gene mutations and polymorphisms. Data were annotated with the Ambry Variant Analyzer tool, including nucleotide and amino acid conservation, biochemical nature of amino acid substitutions, population frequency (Exome Variant Server (National Heart, Lung, and Blood Institute Grand Opportunity Exome Sequencing Project) and the 1000 Genomes Project), and predicted functional impact (including PolyPhen and SIFT in silico prediction tools). Sequence alignments of the reads were viewed using IGV (Integrative Genomics Viewer)	synonymous variants were filtered, except those at the first and last nucleotide position of an exon	The diagnostic rate was significantly higher among families undergoing a trio (37%) as compared to singleton (21%) whole-exome sequencing strategy. Overall, 30.4% (152/500) of patients undergoing WES data anaylsis had a positive gene finding in a characterized gene. Approximately 26% (130/500) received a definitive molecular diagnosis and 4.2% (22/500) received a likely positive result with relevant alterations detected in characterized genes. Among 416 patients who underwent novel gene analysis, 7.5% (31) were positive for a novel gene finding. The overall positive rate among all gene types was 38.5% (160/416). Uncertain findings in characterized genes were found in 8.8% of probands (44/500). Approximately half of all patients (52%) had no relevant gene findings (215/416)	[14]
Analysis of 11,020 *de novo* mutations from whole genomes of 250 trios.	250 parent-child trios (231 trios, 11 families with monozygotic twins and 8 families with dizygotic twins)	Peripheral blood	whole-genome sequencing	Alignment and variant calling were devised on the basis of GATK best practices v2. Sequence data were mapped to the human reference genome Build 37 using bwa 0.5.9-r16, duplicate reads were removed using Picard tools, local indel realignment was performed around indels using GATK IndelRealigner and base qualities were recalibrated using GATK BaseQualityScoreRecalibration. Variants were called using GATK UnifiedGenotyper v1.4 on all samples simultaneously and filtered using GATK VariantQualityScoreRecalibration	Silent mutations were considered. Gene-level mutation rates, separately estimating synonymous, missense and nonsense mutation rates were additionally calculated.	The study shows that *de novo* mutations in the offspring of older fathers are not only more numerous, but also occur more frequently in early-replicating, genic regions. Functional regions exhibit higher mutation rates due to CpG dinucleotides and show signatures of transcription-coupled repair, whereas mutation clusters with a unique signature point to a new mutational mechanism. The data provides a genome-wide mutation rate map for medical and population genetics applications	[80]
Identifictaion of *de novo* mutations by trio analyses in patients with severe intellectual disability (ID)	50 parent-child trios (patients with severe ID and their unaffected parents)	N/A	whole-genome sequencing (average genome-wide coverage of 80-fold)	*De novo* SNVs were identified using complete genomic cgatools cardiff program	not considered	Severe intellectual disability (ID) occurs in 0.5% of newborns. 84 *de novo* SNVs affecting the coding region were identified, which showed a statistically significant enrichment of loss-of-function mutations as well as an enrichment for genes previously implicated in ID-related disorders. These results suggest that *de novo* SNVs and CNVs affecting the coding region are a major cause of severe ID.	[60]
Trio approach to investigate mutational signature and differences between maternally and paternally derived DNMs. A data set of 7,216 autosomal *de novo* mutations of 816 parent-child trios was analyzed	816 parent-child trios	Peripheral blood	whole-genome (average genome-wide coverage of 60-fold)	cgatools calldiff program, to identifiy the parental origin of the DNM allele, phasing of the *de novo* mutations applying a haplotype assembly strategy (HapCompass algorithm), comparison of data sets using data generated by CGI, GATK HaplotypeCaller, PhaseByTransmission, ReadBackedPhasing (99.75% concordance)	not considered	Results show that the number of *de novo* mutations (DNMs) in child increases not only with paternal age, but also with maternal age, and that some genomic regions show enrichment for maternally derived DNMs. Studies of *de novo* mutations have estimated the mutation rate of single-nucleotide variants to be approximately 1 × 10−8 mutations per generation, giving rise to 45–60 DNMs per genome, an average of 45 DNMs per individual was identified. The ratio of the DNMs on the paternal and maternal allelels: 3.6:1, paternally derived DNMs contain higher frequency of T > G and C > A substitution, maternally derived DNMs contain more C > T mutations. Differences between signatures became more pronounced with increasing age of parents at conception, APOBEC-mediated mutations in maternal DNMs. The difference in biology of male and female gamteogenesis gives rise to distinct mutational signatures in children that diverge with increasing parental age	[42]
Identification and analyses of *de novo* mutation in autism spectrum and FMRP-associated genes	343 families (patient with ASD and at least one unaffected sibling)	Peripheral blood	whole-exome sequencing	Standard Illumina analysis pipeline (CASAVA), BWA for alignment, and GATK for refinements, SNV and indel variant caller: Multinomial Model	Silent mutations were considered, proband versus sibling at 40x coverage (53 to 42)	*De novo* small indels and point substitutions come mostly from the paternal line in an age-dependent manner, no significantly greater numbers of *de novo* missense mutations in affected versus unaffected children, but gene-disrupting mutations including nonsense, splice site and frameshifts are twice as frequent (59 to 28), analysis of 350-400 autism susceptibility genes, overall rates of *de novo* mutation: 120 point mutation per genome per generation, 3/4 of new point mutations derive from the father’s germline	[81]
Small insertions and deletions (indels) and large structural variations (SVs) are major contributors to human genetic diversity and disease. Mutation rates and characteristics of de novo indels and SVs in the general population remain largely unexplored	231 parent-child trios (11 quartets with monozygotic (MZ) twins, and eight quartets with dizygotic (DZ) twins, for a total of 258 genetically distinct children)	Peripheral blood	whole-genome, medium coverage (14.5x median sequence depth; 38.4x median physical depth of paired-end sequencing data combined with a family-based design)	Reads were aligned to the GRCh37/hg19 human genome reference using BWA 0.5.9-r164. Aligned data were processed following the Genome Analysis Toolkit (GATK) best practices v2. Duplicate reads were marked using Picard tools (http://picard.sourceforge.net), reads were realigned around indels using GATK IndelRealigner, and base quality scores were recalibrated using GATK BaseRecalibrator.	not considered	This study reports 332 validated *de novo* structural changes identified in whole genomes of 250 families, including complex indels, retrotransposon insertions, and interchromosomal events. The data indicates a mutation rate of 2.94 indels (1–20 bp) and 0.16 SVs ( > 20 bp) per generation. *De novo* structural changes affect on average 4.1 kbp of genomic sequence and 29 coding bases per generation	[82]
Identification of candiate genes for intellectual disability (ID). A meta-analysis on 2.637 *de novo* mutations identified in 2.104 patient-parent trios	2.104 parent-child trios (820 patients, 359 females, 461 males, IQ 50-70 and IQ < 30)	Peripheral blood	whole-exome sequencing (median coverage of 75 × )	Variants were called using GATK unified genotyper (version 3.2-2) and annotated with a custom diagnostic annotation pipeline	not considered	Statistical analyses identified 10 new candiate genes (DLG4, PPM1D, RAC1, SMAD6, SON, SOX5, SYNCRIP, TCF20, TLK2 and TRIP12) that are associated with ID	[83]
Identification of genetic risk factors and the role of *de novo* mutations in autism spectrum disorder (ASD)	175 parent-child trios	N/A	whole-exome sequencing	Data was processed with Picard, BWA for mapping and SNPs were called using GATK for all trios	161 coding region point mutations (101 missense, 50 silent and 10 nonsense mutations)	Half of the patients (46.3%) carried a missense or nonsense *de novo* variant and the overall rate of mutation is only modestly higher than the expected rate. This study provides evidence of CHD8 and KATNAL2 being genuine autism risk factors	[84]
Analysis of 21 *de novo* mutations, 11 were protein altering	20 parent-child trios (individuals with sporadic ASD and their parents)	Peripheral blood	whole-exome sequencing (sufficient coverage to call variants for ~90% of the primary target, 26.4 Mb)	The exome definition was based on consensus coding sequence (CCDS 2009) of the human reference genome (build36). BWA (0.5.6) reads mapping, consensus genotypes were generated using SAMtools, variant positions were pulled and filtered using the samtools.pl varFilter, variants were then run through a custom pipeline, Haystack, to identify Mendelian errors, possibly *de novo* events were then compared against 1,490 other exomes to remove systematic artifacts and rare population variants, variants were then annotated using the SeattleSeq server, possibly *de novo* events were manually inspected using the IGV browser.	not considered	In total, 21 *de novo* mutations were identified, 11 of which were protein altering. Identification of potentially causative *de novo* events in 4 out of 20 probands, particularly among more severely affected individuals, associated genes: FOXP1, GRIN2B, SCN1A and LAMC3. The overall protein-coding *de novo* rate (0.9 events per trio) was slightly higher than expected (0.6 events per trio). The results showed that trio-based whole-exome sequencing is a powerful approach for identifying new candidate genes for ASDs and suggest that *de novo* mutations may contribute substantially to the genetic etiology of ASDs	[9]
Meta-analysis of 6.570 mutations showed that germline methylation influences mutation rates and is increased with paternal age in all families	3 multi-sibling families	Peripheral blood	whole-genome sequencing (24.7-fold coverage on average)	De NovoGear software	not considered	The mutation rate increases with paternal age in all families, but the number of additional mutations per year differed by more than two-fold between families, in parental germline 3.8% of mutations were mosaic, resulting in 1.3% of mutations being shared by siblings, average of 64 DNMs (43-84) per child, the average genome-wide mutation rate of 1.28 × 10−8 mutations per nucleotide per generation and the ratio of paternal to maternal (3.5) mutations are slightly higher but compatible with previous estimates, on average, the number of mutations in the child increases approximately linearly by 2.9 mutations with each additional year in the parents’ age	[85]
The aim of the study was to identify *de novo* variants in individuals with sporadic non-syndromic intellectual disability (ID). The genetic cause of intellectual disability in most patients is still unclear due to the absence of morphological clues, information about the position of such genes, and suitable screening methods.	20 parent-child trios (children with intellectual disability and their parents from ten centres in Germany and Switzerland)	Peripheral blood	whole-exome sequencing (samples were sequenced as 100 bp paired-end runs on a HiSeq2000 system (Illumina). Pools of 12 indexed libraries were sequenced on four lanes)	To identify putative *de novo* variants, read mapping, variant calling, and variant annotation of affected individuals and their parents was performed. To exclude false positives, the identified *de novo* variants were investigated manually with the Integrative Genomics Viewer (IGC).	Silent mutations were considered. The study detected on average 10,500 synonymous and 9,600 non-synonymous variants. The synonymous mutation rate was lower in cases compared to controls, whereas the average number of protein-altering (missense, nonsense, frameshift, and splice site) variants was significantly higher in the case group than in the control group	The study identified 87 *de novo* variants in the case group, with an exomic mutation rate of 1 to 71 per individual per generation. In the control group we identified 24 de novo variants, which is 1·2 events per individual per generation. More participants in the case group had loss-of-function variants than in the control group (20/51 vs 2/20; *p* = 0·022), suggesting their contribution to disease development. 16 patients carried de novo variants in known intellectual disability genes with three recurrently mutated genes (STXBP1, SYNGAP1, and SCN2A)	[10]
Analyses of *de novo* mutations in autism spectrum disorder (ASD)	238 parent-child trios (928 individuals, 225 families (200 quartets, 25 trios))	Peripheral blood	whole-exome sequencing	Short read sequences were aligned to hg18 with BWA, variants were predicted using SAMtools, the data was normalized across each family by only analyzing bases with at least 20 unique reads in all family members, to allow an accurate comparison between the *de novo* burden in probands and siblings the number of *de novo* SNVs found in each sample was divided by the number of bases analyzed (i.e. bases with ≥ 20 unique reads in all familiy members) to calculate a per base rate of *de novo* SNVs	*De novo* silent mutations in all genes, proband versus sibling (29 to 39)	This data demonstrates that non-synonymous *de novo* SNVs, and particularly highly disruptive nonsense and splice-site *de novo* mutations are associated with ASD. Enrichment of non-synonymous and non-sense *de novo* variants in probands relative to sibling control	[11]
*De novo* mutations (DNMs) in neurodevelopmental disorders including intellectual disability, autism spectrum disorder (ASD), schizophrenia. increased risk with advanced paternal age	Healty families participating in the 1000 genome project	N/A	whole-genome and whole-exome sequencing	N/A	*De novo* synonymous SNVs, patients with an ASD vs. healty siblings (0.24 to 0.21)	Whole-genome sequencing: 74 germline SNVs occur *de novo* in an individual’s genome, average human germline SNV mutation rate: 1.18 × 10–8 per position, indel mutation rate has been estimated to be approximately 4 × 10–10 per position (3 *de novo* indels) and 0.02 *de novo* CNVs per genom, Whole-exome: 1 *de novo* mutation per exome, 1 out of 20.563 protein coding genes are hit by a *de novo* mutation per generation	reviewed in ref. [48]
*De novo* mutations may compensate for allele loss due to severely reduced fecundity in common neurodevelopmental and psychiatric diseases, explaining a major paradox in evolutionary genetic theory	10 parent-child trios (patients with unexplained mental retardation)	Peripheral blood	whole-exome sequencing (median coverage of 42-fold)	diBayes algorithm, SOLiD Small Indel Tool	not considered (excluded all nongenic, intronic (other than canonical splice sites) and synonymous variants)	The discovery of nine *de novo* non-synonymous mutations in this cohort of ten affected individuals is concordant with the recently estimated background mutation rate of 0.86 amino-acid–altering mutations per newborn in controls, but it will be important to compare this result to similar data from healthy control trios available	[45]
Evaluating novel bioinformatics approaches to aid identification of new gene-disease associations. Trio analysis to identify both diagnostic genotypes in known genes and candidate genotypes in novel genes	119 parent-child trios	Peripheral blood	whole-exome sequencing (on average, 94.2% of the exome-wide consensus coding sequence was covered with at least 10-fold coverage)	Using BWA-0.5.10, sequencing reads were mapped to a Genome Reference Consortium Human Genome Build 37-derived alignment set including decoy sequences; the same reference genome is used in the 1000 Genomes Project. Polymerase chain reaction duplicates were removed using picard-tools. Single-nucleotide variants and small insertions/deletions (indels) were called using the UnifiedGenotyper of the GATK and annotated using SnpEff-3.3	not analyzed (synonymous mutations were assigned a score of 0)	This study indicates that the application of appropriate bioinformatic approaches to clinical sequence data can also help to implicate novel disease genes and suggest expanded phenotypes for known disease genes.These results suggest that some cases resolved by WES will have direct therapeutic implications on the patient	[12]

Abbreviations: de novo mutations (DNMs), Whole-exome sequencing (WES), Whole-genome sequencing (WGS), autism spectrum disorder (ASD), intellectual disability (ID), amplicon-based deep sequencing (ADS), genome analysis toolkit (GATK), Burrows-Wheeler Aligner (BWA), not applicable (NA)

The detection of germline variants in genes involved in telomere regulation, such as *RTEL1*, *POT1*, *TERC*, and also in the telomerase reverse transcriptase promoter (*TERT*), has been used to identify increased risk of glioma and melanoma, as well as lung, bladder and pancreas cancer [16]. *POT1* germline mutations have been described in high risk families with melanoma [17, 18], colorectal cancer [19], glioma [20], and chronic lymphocytic leukemia [21], and in *KDR* in Hodgkin lymphoma families [22], and demonstrate that family-based NGS approaches work likewise in cancer.

In addition, also in children with metachronous tumors, trio sequencing has unveiled underlying cancer susceptibility [22, 23]. In a recent study performing trio-based whole-exome sequencing in a selected cohort of children with cancer, causative or likely causative pathogenic germline mutations were reported in 20% of the patients. Additionally, in two patients (5%) possible novel cancer-predisposing genes were identified [24]. However, in pediatric oncology, rapid workflows for the use of trio WES in daily clinical routine still need to be established to ensure adaptation of management and treatment in children with inherited CPS such as LFS and DNA repair defects in a timely manner.

The advantage of trio sequencing as compared to sequencing only the affected individual is the leveraging of inheritance information, which enables homozygosity mapping, inference of compound heterozygosity, and the identification of inheritance anomalies [25]. Thus, trio sequencing is of high value in identifying pathogenic variations, but is also important in providing insights into both the inheritance patterns, including *de novo* mutation (DNM) rates, and the pathogenesis of childhood cancer. Noteworthy, some of the mutations identified by trio sequencing might be “private” or rare variants, unique to a single family. These families are faced with the inherent limitation that both the genotype information is limited to one family and likewise the clinical and biological phenotype. Thus, to infer the function of such single (*de novo*) variants, a detailed phenotypic functional characterization is needed to validate whether the disease is conferred by this rare and unique mutation.

## Inheritance

Inherited cancer susceptibility is suspected in families according to well-established criteria [26]. However, owing to phenotypic variability, age-related penetrance, and gender-specific cancer risk, many families with a hereditary CPS will not meet these criteria [27]. Moreover, due to age, genetic background and environmental exposures, mutation processes vary between individuals and families. In addition, some families present with an uninformative pedigree, e.g., due to adoption or a linkage phase that cannot be determined (parents homozygous or parents and child heterozygous for the same alleles).

Over 100 hereditary CPSs have been described so far, the majority with an autosomal dominant inheritance pattern with incomplete penetrance. The most significant CPS with autosomal dominant inheritance is LFS, in which tumor manifestations vary widely within and between families including age of manifestation and cancer type. The reason for this phenomenon still remains elusive.

The minority of CPSs (e.g. most types of Fanconi anemia, many immunodeficiency syndromes) are caused by autosomal recessive inheritance. Homozygosity is particularly relevant in consanguineous families and is often associated with immunodeficiency disorders [28]. Trio sequencing has become a common and successful tool for uncovering underlying genetic defects in these families. In the scenario of compound heterozygous mutations, it also provides important insights into the inheritance patterns (whether the two different mutated recessive alleles of the same gene are transmitted from the mother and father or one originates *de novo*) and, thus, recurrence risk. In addition, in CPSs like Fanconi anemia, trio sequencing also identifies heterozygous carriers who are at increased risk to develop malignancies.

### Concomitant inheritance of two heterozygous mutations in the same CPG pathway

In the majority of children with cancer, the family history is unsuspicious and does not point towards an underlying predisposition syndrome. Since many cancer types in pediatric oncology have been associated with CPSs, this often leads to the clinical scenario of a child presenting with a moderately or highly suspicious cancer type (e.g., osteosarcoma) but an unremarkable family history, raising the question of whether a CPS (e.g., LFS) should be suspected.

In general, LFS patients harbor germline mutations in the *TP53* gene, which predispose to a wide spectrum of early-onset cancer development, including bone and soft tissue sarcomas, brain tumors, breast carcinomas, leukemias, and adrenal cortical carcinomas, and, thus defining the clinical spectrum of LFS and also of the Li-Fraumeni like (LFL) syndrome [8]. Interestingly, in ~25–60% of LFS and LFL patients, a germline *TP53* mutation is not detectable [29]. This suggests the existence of alternative - currently unidentified - or combined mutations in LFS/LFL susceptibility genes. In breast cancer patients, a more severe phenotype has been reported in individuals with double heterozygosity for disease-causing *BRCA1* and *BRCA2* mutations, two genes of the Fanconi anemia/Breast cancer pathway [30].

We previously reported a genetic phenomenon, in which two independent rare germline variants in different genes affecting the same cancer signaling pathway - inherited by the mother and father each - act synergistically in children with cancer [31, 32]. This phenomenon becomes even more complex when more than two SNVs are taken into account or when SNVs are considered together with larger structural alterations, DNA methylation changes and other (epi-)/genetic changes respectively, which are uniquely combined in the particular child with cancer. Thus, we suggest that family-based WES should be complemented by the comprehensive analysis of additional genetic layers. These include mapping technologies allowing the correct genome-wide assessment of large structural variations as well as studies of DNA methylation to detect cancer predisposing syndromes like Beckwith-Wiedemann syndrome which is caused by epimutations which are not detectable by WES or WGS [33].

Taking these observations and hypotheses into account, trio WES of the families of children with cancer offers a unique opportunity to gain further knowledge of the mechanisms of cancer development in children. A recent study on adult sarcoma patients indicates that a striking number of patients—nearly half—harbor putatively pathogenic monogenic and polygenic variation in known and novel cancer genes in the germline [34]. This is of extraordinary significance for children with cancer, since one can speculate that this number must even be higher in children because of currently unrecognized rare variants and genetic interactions between both common and rare variants [34]. For example, only recently, trio sequencing in a family suspicious of an underlying LFS revealed a novel *TP53* mutation in the affected child and mother. Moreover, a nonsense mutation in *ERCC3* inherited by the unaffected father was identified, which might act as possible candidate modifier linked to *TP53* and explain the accelerated tumor onset in the child compared to the mother [35].

However, NGS not only identifies well-known pathogenic variants but likewise a substantial number of variants of unknown significance (VUSs), which are classified as following in a five-tier system for variants relevant to Mendelian disease [(1) benign, (2) likely benign, (3) uncertain significance, (4) likely pathogenic or (5) pathogenic] [36]. Thus, thorough functional validation of these variants is mandatory to correctly classify the VUS as related to the respective condition. Moreover, as there is a complex genotype-to-phenotype association with a complex network of macromolecules (DNA, RNA, proteins) and metabolites linked through physical or biochemical interactions, careful evaluation of the mechanistic impact of identified variants and modifications on molecular interactions such as edgetic perturbation is needed [37, 38].

### De novo mutations

During the last few years, studies related to the role of DNMs that disobey Mendelian inheritance have gained increasing interest, and have shown great potential towards understanding the genetics of human diseases. The main focus of these lies on using trio NGS data, including parents and their children, to determine the properties of and rates at which new mutations appear, which is also of major importance to evolution [39, 40]. DNMs originate post-zygotically or in gametogenesis and result in an embryo with a constitutive mutation [41]. Recent studies propose an - age-dependent -3.9:1 ratio of DNMs on the paternal to the maternal allele, due to the larger number of germline cell divisions in the spermatogenesis compared with the oogenesis [39, 40, 42]. Details on various mutation rates in humans are summarized in Table 2.

**Table 2:** Inherited versus *de novo* mutation rates

	Inherited mutations	De novo mutations
Single-nucleotide variants (SNVs) in the genome	~ 4.4 × 10^6^ ^(1)^	44–82 ^(1, 2)^
SNVs in the exome (coding SNVs)	22,186 ^(2)^	1–2 ^(3)^
Small insertion and deletions (INDELs)	~550,000 ^(1)^	up to 9 ^(4)^
Copy number variations (CNVs)	~276 ^(1, 2)^	0.0077–0.041 ^(4)^
Ratio of paternal allele versus maternal allele	1 : 1^(5, 6)^	3.5–3.9: 1 ^(5, 6,7)^
Parental age effect at conception	No effect^(8)^	Strong effect^(8)^

An increasing number of studies suggest that DNMs are of particular importance in conditions such as neurodevelopmental diseases and rare sporadic malformation syndromes, including severe congenital heart disease [9, 11, 43–45]. As a result of less stringent evolutionary selection, DNMs are commonly more deleterious than inherited variations and contribute to the persistence of early-onset lethal diseases in the population [46–48].

Despite these important insights and their far-reaching implications for causes, mechanisms and preventive strategies in childhood cancer and counseling of affected families, the contribution of germline DNMs to the disease burden in childhood cancer is almost completely unexplored and, thus, the risk of recurrence in future children of the parents. Paying particular attention to DNMs using family-based WES/WGS approaches is crucial to further identifying cancer predisposition in children. In particular, one could speculate that DNMs play a fundamental role in the development of congenital and early-onset cancer in children as well as in families without a cancer history. This is underlined by recent epidemiological studies indicating that older parental age is associated with pediatric cancer risk in the offspring [49].

## **Post-zygoticde novomutations and low-level parental mosaicism**

Post-zygotical appearance of DNMs can lead to embryonic mosaicism. For genetic counselling, it is crucial to distinguish between post-zygotical DNMs and true heterozygous mutations. As such, it has been proposed that DNMs with an allelic ratio below 32.8% for WGS, 39.3% for amplicon-based deep sequencing, and 33.9% for Sanger sequencing might reflect mosaic mutations, as they significantly deviate from the statistically expected ratio for true heterozygous mutations [50]. However, these numbers are prone to inter-laboratory variation. Acuna-Hidalgo et al. reported that an important fraction of presumed germline DNMs indeed occurred either post-zygotically or as a consequence of low-level mosaicism in one of the parents. In addition, these data suggest that each individual carries at least two to seven DNMs of post-zygotic origin [50].

Gonadal mosaicism contributes to the recurrence of disorders in a seemingly *de novo* manner and, thus, transmission of disease-causing mutations from an unaffected parent [51]. A recent study indicates that gonadal mosaicism for disease-causing CNVs is not restricted to germ cells, but can be carried as low-level mosaicism in the blood of unaffected parents [52].

The clinical phenotype caused by DNMs is determined by the proportion of affected cells and the type of tissues involved, both of which critically depend on the time of the occurrence of the mutation [50]. In developmental disorders, post-zygotic DNMs are receiving more and more attention as significant contributors to disease evolution [41].

Mutations appear from early embryogenesis throughout adult life, leading to a high prevalence of mosaicism for SNVs; however, to date, the range of such mosaicism remains unclear [50]. Therefore, it is important to discriminate technical artifacts from biologically relevant allele imbalances, and to differentiate between post-zygotic and germline DNMs [50].

In summary, some DNMs previously presumed to be germline actually occur either post-zygotically in the child or are inherited from low-level mosaicism in one of the parents. This might have important clinical implications in pediatric oncology. It could be hypothesized that in childhood cancer this proportion is at least as high as the reported 6.5% for DNMs, which underlines the importance of identification. Pathogenic variants in CPGs in the mosaic state influence the risk of recurrence in seemingly sporadic cancers caused by DNMs, and, thus, accurate genetic counseling of affected families.

Notably, Conrad et al. reported important differences in the proportion of CpG mutations, the ratio of transitions and transversions, the clonality of mutations, their occurrence at sites under selective constraint, and the evidence for transcription-coupled repair in germline, non-germline and inherited DNMs [53].

## Parental bias and age effects

Previous studies indicated that advanced parental age might be associated with a higher incidence of children with cancer [54]. A study from Sweden demonstrated a 25% increased cancer risk of brain tumors in children with fathers over the age of 30, compared to fathers, who were younger than 25 years [55]. In general, childhood leukemia has a risk of 1 in 25 000, whereas this rate increases due to advanced paternal age up to 1 in 17 000 (reviewed in [56]). However, a potential effect of the parental – particularly the paternal - age on cancer incidence and type in children needs to be further elucidated in more detail.

Moreover recent studies suggest that mutations in particular genes (e.g. *FGFR2*, *FGFR3*, *HRAS*, and *PTPN11*) confer growth advantages to spermatogonial cells, leading to autosomal dominant disorders such as Apert syndrome and achondroplasia [39, 57]. This is particularly interesting because cell growth, differentiation, cycle, and cell senescence are crucial to normal development, and are essentially regulated by the Ras/mitogen activated protein kinase (MAPK) pathway. Germline mutations in genes of the Ras/MAPK pathway cause the so-called “RASopathies”, developmental disorders which predispose to malignancies including leukemia, central nervous system and extracranial solid tumors (reviewed in [58]). In addition, germline and somatic *FGFR1* mutations and MAPK-ERK pathway activation play a key event of many developmental disorders of the brain such as the dysembryoplastic neuroepithelial tumor. Therefore, germline analysis of *FGFR1* is recommended in both familial cases and selected sporadic tumors (e.g. multinodular growth) [59].

## Types of aberrations

### Single-nucleotide and copy number variants

By trio sequencing, Gilissen et al. demonstrated that *de novo* SNVs and copy number variants (CNVs) in coding regions are an important cause of severe ID in an extensively pre-studied genetic cohort [60]. Recently, a mosaic RAS pathway gene aberration, a large *SOS1* duplication, was reported in a child with features of Noonan syndrome and early-onset rhabdomyosarcoma [61]. Additionally, an approximately 5.8 Mbp 14q32.13q32.2 germline deletion of the DICER1 locus was also lately reported in a child with multiple DICER1 syndrome related tumors, including a small lung cyst, a ciliary body medulloepithelioma, and a pediatric cystic nephroma [62]. This likewise highlights the need to further elucidate the role of germline *de novo* SNVs and CNVs in childhood cancer.

In LFS, there is an unexplained individual disparity between tumor patterns and ages among subjects and families in which cancer onset accelerates with successive generations [63]. It has been speculated that, in *TP53* haploinsufficiency, anticipation is caused by accumulation of CNVs [63]. Although, in a WGS study of two generations of LFS kindreds, Arrifin et al. could not demonstrate an association of *de novo* or total CNVs with the phenotype of LFS. Instead, they proposed a model in which constitutive resistance to tumorigenesis is attenuated by variants from non-carrier parents in the child with late cancer onset [64]. This, once more, strengthen our hypothesis that inherited monoallelic germline mutations in more than one CPG might contribute to a substantial proportion of childhood cancers.

However, while WES provides a highly accurate way to obtain SNVs, the high level of noise and biases in WES data limit CNV detection with current detection tools for WES data. Consequently, in selected families, array analysis might additionally be used to obtain CNV information.

### Synonymous mutations

Out of the 10,000 mutations in the *TP53* gene that have been reported in the International Agency for Research on Cancer tumor database, about 4.1% are synonymous [65]. Over the last decade, silent mutations have been described in more than 40 diseases (genes) (e.g. in familial adenomatous polyposis (*APC*), ataxia telangiectasia (*ATM*), neurofibromatosis type 1 (*NF1*), and hereditary non-polyposis colorectal cancer (*MLH1*)), and may also play a role in tumorigenesis [66]. Additional studies indicated synonymous mutations in genes like *CFTR*, *TCOF1*, *WT1*, *EGFR*, *IRGM*, *NTF3*. These genes are directly associated with several human diseases, including non-small-cell lung carcinoma and immune diseases [67].

### Mutations in the untranslated region and in regulatory elements

Genetic variations in the mRNA untranslated regions (UTRs) might disrupt the motifs of the UTR, influence cancer development and the malignant phenotype of cancer cells [68]. Mutations in the UTR have been associated with susceptibility to diseases such as breast cancer [69].

Telomerase reverse transcriptase (*TERT*) promoter mutations lead to telomerase activation and cell-cycle progression. There is mounting evidence, that telomerase not only plays a critical role in cellular senescence but likewise in carcinogenesis. Recently, *TERT* mutations including hot spot mutations in the regulatory region of the gene have been described in various malignancies and have also been linked to poor prognosis [70].

## Uniparental disomy

Uniparental disomies (UPDs) have been reported to be associated with imprinting disorders [71], recessive disease [72], ID [73], and trisomy mosaicism [74], as well as being a contributor to rare genetic diseases. For example, the imprinting disorder Beckwith-Wiedemann syndrome can - among other mechanisms - be caused by UPD11 and is related to an increased risk of cancer development in childhood [75]. In addition, the development of certain subtypes of nephroblastoma has been described as being based on alterations at imprinted loci [76]. King et al. implemented a method for detecting UPDs in trios [25]. However, whereas UPD can be captured by WES/WGS, this is not the case for epimutations causing imprinting disorders. Thus, epigenetic alterations contributing to specific phenotype-epigenotype/genotype correlations implicating different recurrence risks additionally need to be excluded by dedicated epigenetic technologies.

## Disclosing a hereditary CPS by trio sequencing

Identifying children with hereditary CPS by trio germline sequencing has far-reaching consequences. (Table 3) For example in CMMRD, the parents may – as still being young - so far be asymptomatic carriers of the genetic alterations and, thus, likewise be affected by Lynch syndrome. Disclosing the hereditary CPS in the parents may be clinically highly important by means of initiating early cancer surveillance protocols on one hand [77]. On the other hand, it may constitute an enormous life-long psychological distress, and could have a deep effect on quality of life and family planning.

**Table 3:** Benefits of trio germline sequencing in children with cancer

	Sequencing of the index patient only	Trio sequencing
Identification of well-known CPSs	+	+
SNVs, indels, SVs, CNVs	+	+
Inheritance information including		
Homozygosity mapping	Isodisomy	+
Inference of compound heterozygosity	−	+
Inheritance anomalies	−	+
De novo mutations incl. age effects	−	+
Mosaicism	(+)	+
Concomitant variants	+	+
Phenotypic variability, age-related penetrance and gender-specific cancer risk	−	+
Phasing of variants	−	+
Treatment adaptation & surveillance	+	+
Risk evaluation of unaffected parents, surveillance & precision prevention	(−)	+
Determination of the accurate risk to carry the variant for other family members	−	+
Prenatal diagnostics	n/a	+

In contrast to testing the index patient only, trio sequencing discloses inheritance patterns and, thus, might put the psychological burden of inheritance to the transmitting parent. In addition, testing for siblings at-risk has to be discussed, balancing the pros and cons including disease onset in childhood and the right to decide autonomously on predictive testing.

## Concluding remarks

This review underlines the power of and the need for comprehensive parent-child NGS analyses of pediatric cancer families. Such analyses have the potential to reveal a striking new landscape of inheritance in childhood cancer by identifying pathogenic heterozygous and homozygous mutations, concomitant heterozygous mutations in the same CPG pathway, *de novo* mutations, and parental mosaicism, with important implications beyond only the affected child. Consequently, we recommend to routinely integrate trio germline sequencing into pediatric oncology by offering it to each family with a child newly diagnosed with cancer. However, trio sequencing will reveal numerous variants of unknown significance for which thorough functional validation is mandatory but remains challenging.

Beyond that—based on a parent-child approach—future research is required to elucidate the clinical implications of non-Mendelian inheritance, the complex interactions between genetic predisposition and environmental factors, and the genetic and epigenetic interplay. This will give important insights into the pathogenesis of cancer in childhood and the complex genotype-to-phenotype association in most CPSs^78^.

In addition, by international efforts with large-scaled studies, evidence-based clinical surveillance protocols with the aim of early tumor detection and reduction of cancer and treatment-related morbidity and mortality need to be established. Moreover, future studies are needed to investigate the needs and preferences of affected families and the psychological long-term impact of the burden of knowing.

## References

[1] Martin-Lorenzo A, Hauer J, Vicente-Duenas C, Auer F, Gonzalez-Herrero I, Garcia-Ramirez I (2015). Infection exposure is a causal factor in B-cell precursor acute lymphoblastic leukemia as a result of Pax5-inherited susceptibility. Cancer Discov.

[2] Zhang J, Walsh MF, Wu G, Edmonson MN, Gruber TA, Easton J (2015). Germline mutations in predisposition genes in pediatric cancer. N Eng J Med.

[3] Waszak SM, Tiao G, Zhu B, Rausch T, Muyas F, Rodriguez-Martin B, et al. Germline determinants of the somatic mutation landscape in 2,642 cancer genomes. bioRxiv. 2017. 10.1101/208330.

[4] Brozou T, Taeubner J, Velleuer E, Dugas M, Wieczorek D, Borkhardt A (2017). Genetic predisposition in children with cancer - affected families’ acceptance of Trio-WES. Eur J Pediatr.

[5] Bardai A, Overwater E, Aalfs CM (2016). Germline mutations in predisposition genes in pediatric cancer. N Eng J Med.

[6] Renaux-Petel M, Charbonnier F, Thery JC, Fermey P, Lienard G, Bou J, et al. Contribution of de novo and mosaic TP53 mutations to Li-Fraumeni syndrome. J Med Genet. 2017;55:173–80. 10.1136/jmedgenet-2017-104976 29070607

[7] Kresak JL, Walsh M (2016). Neurofibromatosis: a review of NF1, NF2, and Schwannomatosis. J Pediatr Genet.

[8] Kratz CP, Achatz MI, Brugieres L, Frebourg T, Garber JE, Greer MC (2017). Cancer screening recommendations for individuals with Li-Fraumeni syndrome. Clin Cancer Res.

[9] O’Roak BJ, Deriziotis P, Lee C, Vives L, Schwartz JJ, Girirajan S (2011). Exome sequencing in sporadic autism spectrum disorders identifies severe de novo mutations. Nat Genet.

[10] Rauch A, Wieczorek D, Graf E, Wieland T, Endele S, Schwarzmayr T (2012). Range of genetic mutations associated with severe non-syndromic sporadic intellectual disability: an exome sequencing study. Lancet.

[11] Sanders SJ, Murtha MT, Gupta AR, Murdoch JD, Raubeson MJ, Willsey AJ (2012). De novo mutations revealed by whole-exome sequencing are strongly associated with autism. Nature.

[12] Zhu X, Petrovski S, Xie P, Ruzzo EK, Lu YF, McSweeney KM (2015). Whole-exome sequencing in undiagnosed genetic diseases: interpreting 119 trios. Genet Med: Off J Am Coll Med Genet.

[13] Lee H, Deignan JL, Dorrani N, Strom SP, Kantarci S, Quintero-Rivera F (2014). Clinical exome sequencing for genetic identification of rare Mendelian disorders. JAMA.

[14] Farwell KD, Shahmirzadi L, El-Khechen D, Powis Z, Chao EC, Tippin Davis B (2015). Enhanced utility of family-centered diagnostic exome sequencing with inheritance model-based analysis: results from 500 unselected families with undiagnosed genetic conditions. Genet Med.

[15] Mestek-Boukhibar L, Clement E, Jones WD, Drury S, Ocaka L, Gagunashvili A, et al. Rapid Paediatric Sequencing (RaPS): comprehensive real-life workflow for rapid diagnosis of critically ill children. J Med Genet. 2018. 10.1136/jmedgenet-2018-105396. 10.1136/jmedgenet-2018-105396 PMC6252361 30049826

[16] Bell RJ, Rube HT, Xavier-Magalhaes A, Costa BM, Mancini A, Song JS (2016). Understanding TERT promoter mutations: a common path to immortality. Mol Cancer Res: MCR.

[17] Robles-Espinoza CD, Harland M, Ramsay AJ, Aoude LG, Quesada V, Ding Z (2014). POT1 loss-of-function variants predispose to familial melanoma. Nat Genet.

[18] Shi J, Yang XR, Ballew B, Rotunno M, Calista D, Fargnoli MC (2014). Rare missense variants in POT1 predispose to familial cutaneous malignant melanoma. Nat Genet.

[19] Chubb D, Broderick P, Dobbins SE, Frampton M, Kinnersley B, Penegar S (2016). Rare disruptive mutations and their contribution to the heritable risk of colorectal cancer. Nat Commun.

[20] Bainbridge MN, Armstrong GN, Gramatges MM, Bertuch AA, Jhangiani SN, Doddapaneni H (2015). Germline mutations in shelterin complex genes are associated with familial glioma. J Natl Cancer Inst.

[21] Speedy HE, Kinnersley B, Chubb D, Broderick P, Law PJ, Litchfield K, et al. Germline mutations in shelterin complex genes are associated with familial chronic lymphocytic leukemia. Blood. 2016;128:2319–26. 10.1182/blood-2016-01-695692 PMC5271173 27528712

[22] Rotunno M, McMaster ML, Boland J, Bass S, Zhang X, Burdett L (2016). Whole exome sequencing in families at high risk for Hodgkin lymphoma: identification of a predisposing mutation in the KDR gene. Haematologica.

[23] Fremerey J, Balzer S, Brozou T, Schaper J, Borkhardt A, Kuhlen M. Embryonal rhabdomyosarcoma in a patient with a heterozygous frameshift variant in the DICER1 gene and additional manifestations of the DICER1 syndrome. Fam Cancer. 2016;6:401–5. 10.1007/s10689-016-9958-5 27896549

[24] Diets IJ, Waanders E, Ligtenberg MJ, van Bladel DAG, Kamping EJ, Hoogerbrugge PM (2018). High yield of pathogenic germline mutations causative or likely causative of the cancer phenotype in selected children with cancer. Clin Cancer Res.

[25] King DA, Fitzgerald TW, Miller R, Canham N, Clayton-Smith J, Johnson D (2014). A novel method for detecting uniparental disomy from trio genotypes identifies a significant excess in children with developmental disorders. Genome Res.

[26] Knapke S, Zelley K, Nichols KE, Kohlmann W, Schiffman JD. Identification, management, and evaluation of children with cancer-predisposition syndromes. Am Soc Clin Oncol Educ Book. 2012:576–84. 10.14694/EdBook_AM.2012.32.8 24451799

[27] Nagy R, Sweet K, Eng C (2004). Highly penetrant hereditary cancer syndromes. Oncogene.

[28] Hadizadeh H, Salehi M, Khoramnejad S, Vosoughi K, Rezaei N (2017). The association between parental consanguinity and primary immunodeficiency diseases: A systematic review and meta-analysis. Pediatr Allergy Immunol.

[29] McBride KA, Ballinger ML, Killick E, Kirk J, Tattersall MH, Eeles RA (2014). Li-Fraumeni syndrome: cancer risk assessment and clinical management. Nat Rev Clin Oncol.

[30] Heidemann S, Fischer C, Engel C, Fischer B, Harder L, Schlegelberger B (2012). Double heterozygosity for mutations in BRCA1 and BRCA2 in German breast cancer patients: implications on test strategies and clinical management. Breast Cancer Res Treat.

[31] Kuhlen M, Borkhardt A. Trio sequencing in pediatric cancer and clinical implications. EMBO Mol Med. 2018;10:e8641. 10.15252/emmm.201708641 PMC5887902 29507082

[32] Taeubner J, Brozou T, Qin N, Bartl J, Ginzel S, Schaper J, et al. Congenital embryonal rhabdomyosarcoma caused by heterozygous concomitant PTCH1 and PTCH2 germline mutations. Eur J Hum Genet. 2017;26:137–42. 10.1038/s41431-017-0048-4 PMC5839031 29230040

[33] Korbel JO, Urban AE, Affourtit JP, Godwin B, Grubert F, Simons JF (2007). Paired-end mapping reveals extensive structural variation in the human genome. Science.

[34] Ballinger ML, Goode DL, Ray-Coquard I, James PA, Mitchell G, Niedermayr E (2016). Monogenic and polygenic determinants of sarcoma risk: an international genetic study. Lancet Oncol.

[35] Franceschi S, Spugnesi L, Aretini P, Lessi F, Scarpitta R, Galli A (2017). Whole-exome analysis of a Li-Fraumeni family trio with a novel TP53 PRD mutation and anticipation profile. Carcinogenesis.

[36] Richards S, Aziz N, Bale S, Bick D, Das S, Gastier-Foster J (2015). Standards and guidelines for the interpretation of sequence variants: a joint consensus recommendation of the American College of Medical Genetics and Genomics and the Association for Molecular Pathology. Genet Med.

[37] Betts MJ, Lu Q, Jiang Y, Drusko A, Wichmann O, Utz M (2015). Mechismo: predicting the mechanistic impact of mutations and modifications on molecular interactions. Nucleic Acids Res.

[38] Zhong Q, Simonis N, Li QR, Charloteaux B, Heuze F, Klitgord N (2009). Edgetic perturbation models of human inherited disorders. Mol Syst Biol.

[39] Campbell CD, Eichler EE (2013). Properties and rates of germline mutations in humans. Trends Genet.

[40] Kong A, Frigge ML, Masson G, Besenbacher S, Sulem P, Magnusson G (2012). Rate of de novo mutations and the importance of father’s age to disease risk. Nature.

[41] Acuna-Hidalgo R, Veltman JA, Hoischen A (2016). New insights into the generation and role of de novo mutations in health and disease. Genome Biol.

[42] Goldmann JM, Wong WS, Pinelli M, Farrah T, Bodian D, Stittrich AB (2016). Parent-of-origin-specific signatures of de novo mutations. Nat Genet.

[43] Deciphering Developmental Disorders Study. (2017). Prevalence and architecture of de novo mutations in developmental disorders. Nature.

[44] Ng SB, Bigham AW, Buckingham KJ, Hannibal MC, McMillin MJ, Gildersleeve HI (2010). Exome sequencing identifies MLL2 mutations as a cause of Kabuki syndrome. Nat Genet.

[45] Vissers LE, de Ligt J, Gilissen C, Janssen I, Steehouwer M, de Vries P (2010). A de novo paradigm for mental retardation. Nat Genet.

[46] Crow JF (2000). The origins, patterns and implications of human spontaneous mutation. Nat Rev Genet.

[47] Eyre-Walker A, Keightley PD (2007). The distribution of fitness effects of new mutations. Nat Rev Genet.

[48] Veltman JA, Brunner HG (2012). De novo mutations in human genetic disease. Nat Rev Genet.

[49] Wang R, Metayer C, Morimoto L, Wiemels JL, Yang J, DeWan AT (2017). Parental age and risk of pediatric cancer in the offspring: a population-based record-linkage study in California. Am J Epidemiol.

[50] Acuna-Hidalgo R, Bo T, Kwint MP, van de Vorst M, Pinelli M, Veltman JA (2015). Post-zygotic point mutations are an underrecognized source of de novo genomic variation. Am J Hum Genet.

[51] Natacci F, Baffico M, Cavallari U, Bedeschi MF, Mura I, Paffoni A (2008). Germline mosaicism in achondroplasia detected in sperm DNA of the father of three affected sibs. Am J Med Genet A.

[52] Campbell IM, Yuan B, Robberecht C, Pfundt R, Szafranski P, McEntagart ME (2014). Parental somatic mosaicism is underrecognized and influences recurrence risk of genomic disorders. Am J Hum Genet.

[53] Conrad DF, Keebler JE, DePristo MA, Lindsay SJ, Zhang Y, Casals F (2011). Variation in genome-wide mutation rates within and between human families. Nat Genet.

[54] Johnson KJ, Carozza SE, Chow EJ, Fox EE, Horel S, McLaughlin CC (2009). Parental age and risk of childhood cancer: a pooled analysis. Epidemiol (Camb, Mass).

[55] Hemminki K, Kyyronen P, Vaittinen P (1999). Parental age as a risk factor of childhood leukemia and brain cancer in offspring. Epidemiol (Camb, Mass).

[56] Conti SL, Eisenberg ML (2016). Paternal aging and increased risk of congenital disease, psychiatric disorders, and cancer. Asian J Androl.

[57] Goriely A, Wilkie AO (2012). Paternal age effect mutations and selfish spermatogonial selection: causes and consequences for human disease. Am J Hum Genet.

[58] Rauen KA (2013). The RASopathies. Annu Rev Genom Hum Genet.

[59] Rivera B, Gayden T, Carrot-Zhang J, Nadaf J, Boshari T, Faury D (2016). Germline and somatic FGFR1 abnormalities in dysembryoplastic neuroepithelial tumors. Acta Neuropathol.

[60] Gilissen C, Hehir-Kwa JY, Thung DT, van de Vorst M, van Bon BW, Willemsen MH (2014). Genome sequencing identifies major causes of severe intellectual disability. Nature.

[61] Salem B, Hofherr S, Turner J, Doros L, Smpokou P (2016). Childhood rhabdomyosarcoma in association with a RASopathy clinical phenotype and mosaic germline SOS1 duplication. J Pediatr Hematol Oncol.

[62] de Kock L, Geoffrion D, Rivera B, Wagener R, Sabbaghian N, Bens S, et al. Multiple DICER1-related tumors in a child with a large interstitial 14q32 deletion. Genes Chromosomes Cancer. 2018;57:223–30. 10.1002/gcc.22523 29315962

[63] Shlien A, Tabori U, Marshall CR, Pienkowska M, Feuk L, Novokmet A (2008). Excessive genomic DNA copy number variation in the Li-Fraumeni cancer predisposition syndrome. Proc Natl Acad Sci USA.

[64] Ariffin H, Hainaut P, Puzio-Kuter A, Choong SS, Chan AS, Tolkunov D (2014). Whole-genome sequencing analysis of phenotypic heterogeneity and anticipation in Li-Fraumeni cancer predisposition syndrome. Proc Natl Acad Sci USA.

[65] Strauss BS (2000). Role in tumorigenesis of silent mutations in the TP53 gene. Mutat Res.

[66] Chamary JV, Parmley JL, Hurst LD (2006). Hearing silence: non-neutral evolution at synonymous sites in mammals. Nat Rev Genet.

[67] Sauna ZE, Kimchi-Sarfaty C (2011). Understanding the contribution of synonymous mutations to human disease. Nat Rev Genet.

[68] Vislovukh A, Vargas TR, Polesskaya A, Groisman I (2014). Role of 3’-untranslated region translational control in cancer development, diagnostics and treatment. World J Biol Chem.

[69] Dorairaj JJ, Salzman DW, Wall D, Rounds T, Preskill C, Sullivan CA (2014). A germline mutation in the BRCA1 3’UTR predicts Stage IV breast cancer. BMC Cancer.

[70] Schwaederle M, Krishnamurthy N, Daniels GA, Piccioni DE, Kesari S, Fanta PT, et al. Telomerase reverse transcriptase promoter alterations across cancer types as detected by next-generation sequencing: A clinical and molecular analysis of 423 patients. Cancer. 2017;124:1288–96. 10.1002/cncr.31175 PMC5839978 29211306

[71] Yamazawa K, Ogata T, Ferguson-Smith AC (2010). Uniparental disomy and human disease: an overview. Am J Med Genet C Semin Med Genet.

[72] Zlotogora J (2004). Parents of children with autosomal recessive diseases are not always carriers of the respective mutant alleles. Hum Genet.

[73] Papenhausen P, Schwartz S, Risheg H, Keitges E, Gadi I, Burnside RD (2011). UPD detection using homozygosity profiling with a SNP genotyping microarray. Am J Med Genet A.

[74] Kotzot D (2008). Complex and segmental uniparental disomy updated. J Med Genet.

[75] Mussa A, Molinatto C, Baldassarre G, Riberi E, Russo S, Larizza L (2016). Cancer Risk in Beckwith-Wiedemann Syndrome: a systematic review and meta-analysis outlining a novel (epi)genotype specific histotype targeted screening protocol. J Pediatr.

[76] Cerrato F, Sparago A, Verde G, De Crescenzo A, Citro V, Cubellis MV (2008). Different mechanisms cause imprinting defects at the IGF2/H19 locus in Beckwith-Wiedemann syndrome and Wilms’ tumour. Hum Mol Genet.

[77] Villani A, Tabori U, Schiffman J, Shlien A, Beyene J, Druker H (2011). Biochemical and imaging surveillance in germline TP53 mutation carriers with Li-Fraumeni syndrome: a prospective observational study. Lancet Oncol.

[78] Taeubner J, Wieczorek D, Yasin L, Brozou T,Borkhardt A, Kuhlen M (2018) Penetrance and Expressivity in Inherited Cancer Predisposing Syndromes. Trends in Cancer: 10.1016/j.trecan.2018.09.00. 10.1016/j.trecan.2018.09.002 30352675

[79] Besenbacher S, Liu S, Izarzugaza JM, Grove J, Belling K, Bork-Jensen J, et al. Novel variation and de novo mutation rates in population-wide de novo assembled danish trios. Nat Commun. 2015;6:5969. 10.1038/ncomms6969 PMC4309431 25597990

[80] Francioli LC, Polak PP, Koren A, Menelaou A, Chun S, Renkens I, et al. Genome-wide patterns and properties of de novo mutations in humans. Nat Genet. 2015;47:822–6. 10.1038/ng.3292 PMC4485564 25985141

[81] Iossifov I, Ronemus M, Levy D, Wang Z, Hakker I, Rosenbaum J, et al. De novo gene disruption in children on the autistic spectrum. Neuron. 2012;74:285–99. 10.1016/j.neuron.2012.04.009 PMC3619976 22542183

[82] Kloosterman WP, Francioli LC, Hormozdiari F, Marschall T, Hehir-Kwa JY, Abdellaoui A, et al. Characteristics of de novo structural changes in the human genome. Genome Res. 2015;25:792–801. 10.1101/gr.185041.114 PMC4448676 25883321

[83] Lelieveld SH, Reijnders MR, Pfundt R, Yntema HG, Kamsteeg EJ, de Vries P, et al.Meta-analysis of 2,104 trios provides support for 10 new genes for intellectual disability. Nat Neurosci. 2016;19:1194–6. 10.1038/nn.4352 27479843

[84] Neale BM, Kou Y, Liu L, Ma'ayan A, Samocha KE, Sabo A, et al. Patterns and rates of exonic de novo mutations in autism spectrum disorders. Nature. 2013;485:242–5. 10.1038/nature11011 PMC3613847 22495311

[85] Rahbari R, Wuster A, Lindsay SJ, Hardwick RJ, Alexandrov LB, Turki SA, et al. Timing, rates and spectra of human germline mutation. Nat Genet. 2016;48:126–33. 10.1038/ng.3469 PMC4731925 26656846

